# Effects of novel bioorganic fertilizer application on soil enzymes and bacterial community in multi-site rice paddies in China

**DOI:** 10.1186/s13568-021-01241-5

**Published:** 2021-05-31

**Authors:** Zuren Li, Jincai Han, Haodong Bai, Di Peng, Lifeng Wang, Lianyang Bai

**Affiliations:** 1 Hunan Provincial Key Laboratory for Biology and Control of Weeds, Hunan Academy of Agricultural Sciences, Changsha, 410125 China; 2 Collaborative Innovation Center for Field Weeds Control, Hunan University of Humanities, Science and Technology, Loudi, 417000 Hunan China

**Keywords:** Bioorganic fertilizer, Rice paddy, Weed management, Bacterial community, Soil enzyme

## Abstract

Application of a novel bioorganic fertilizer (BIO) has been effectively used to inhibit weeds in rice paddies. To identify changes in soil bacterial community and enzymes in response to BIO treatments, field experiments were carried out in five major rice-growing areas in China. The dominant phylogenetic groups recorded included *Proteobacteria*, *Actinobacteria*, *Bacteroidetes*, *Firmicutes* and *Acidobacteria*. *Anaeromyxobacter*, *Bacteroides*, *Bifidobacterium*, *Escherichia- Shigella*, *Geobacter* and *Haliangium* were significantly different between BIO-treatment and untreated control and aided in general function (R), amino acid transport, metabolism (E) and transcription (K) clusters. The soil chemical properties and enzyme activities were less affected by BIO at these study sites. RDA analysis showed that soil bacterial community had a significant positive correlations among northern latitude, eastern longitude, exchangeable K, total K, total P, soil pH, and total N, except for organic matter, hydrolytic N and extractable P. Overall, our work showed that application of BIO does not alter the main community structure and functional diversity of soil bacteria in rice paddies and should be encouraged for use as a sustainable weed management strategy.

**Supplementary Information:**

The online version contains supplementary material available at 10.1186/s13568-021-01241-5.

## Introduction

Soils provide the physical anchor for crops and therefore the management of its quality is key, in order to maintain agricultural productivity and ecosystems sustainability (Bardgett 2010). Soil bacterial community and enzyme activities are important components for soil functioning. Soil bacterial communities are essential part of the microbial community, and play roles in soil habitats in terms of their biodiversity and biomass (Bahram et al. 2018). Microbial communities play specific roles in biogeochemical cycling and soil functioning through traits such as their species richness, biotic interactions and decomposing activities (Kumar et al. 2018). Soil enzyme activities are reliable parameters for monitoring both positive and negative effects of agro-ecosystems on soil biological activity and crop productivity of agro-ecosystems (Stark et al. 2008). Data on soil enzyme activity include the direct or indirect expression of the soil physio-chemical properties, microorganism, above-ground plants, disturbance and evolution (Zhu et al. 2019). Both changes in soil bacterial community and enzyme activities can affect ecosystem stability in relation to environmental conditions. Thus, the assessment of the influence of biotic and abiotic stressors on the soil microbial community and enzyme activities has become a hot research area for the sustainability of agricultural ecosystem.

To protect rice against weeds, the application of synthetic herbicides is by far the most common management strategy followed. Evaluating the negative effects of herbicides on soil functions is critical for the sustainable utilization of soil and prevention of damage to agricultural ecosystems (Carvalho 2017). For instance, it was shown that treatment of soil with glyphosate at a rate of 360 g a.i. ha^−1^, had no significant effect on the soil enzymes (Cherni et al. 2015). Nguyen et al. (2018) showed that effects on soil enzymes were lower in light-textured tenosol soil than vertosol and chromosol soils under glyphosate or Roundup CT application. Also, it was reported that the soil microbial structure of irrigated soils under herbicide (triasulfuron and prosulfocarb) treatment exhibited a higher proportion of *Actinobacteria* and lower relative amount of fungi than non-irrigated soils (Delgado et al. 2019). After bispyribac sodium (35 g ha^−1^ and 70 g ha^−1^) application, the soil microbial biomass carbon, dehydrogenase, alkaline phosphatase and urease activities were significantly decreased, and the heterotrophic bacteria, actinomycetes and fungal population also declined (Kumar et al. 2020). Therefore, the soil type and herbicide formulation are important factors that influence soil functions (Borowik et al. 2017).

Bioorganic fertilizers are mostly organic fertilizer obtained by secondary fermentation involving particular microorganisms (Ling et al. 2012). Bioorganic fertilizers improve soil vitality and organic matter content, and strengthen the effectiveness of pest biocontrol agents (Barakat and Al-Masri 2009). A novel bioorganic fertilizer developed by Wu et al. (2014) showed that it had the potential to inhibit bacterial wilt and suppress *Ralstonia solanacearum* growth. The early application of urea ammonium nitrate (UAN 28%-N) at 112 kg/ha was shown to increase the emergence rate of certain weeds such as *Chenopodium album*, *Polygonum persicaria*, *Seteria faberi*, and *Abutilon theophrasti* (Pearson et al. 2008)*.* The application of pig manure reduced the effects of applying NPK complex fertilizers during the heading and ripening stages in rice, on paddy bacterial communities, but the effects of straw returning was not obvious (Wang et al. 2019a, b). Hence, bioorganic fertilizer is not only a fundamental soil quality resource, but also an effective carrier for the biocontrol of pests.

In our previous study, we developed a novel bioorganic weeding fertilizer (BIO) by fermenting mature compost with kitchen garbage, maize straw, wood-destroying fungal dregs, rice straw, tobacco straw, plant ash, chicken, and sheep manure. The novel BIO was found to be effective in controlling grass and broad-leaved weeds in three rice fields (Huanan, Hainan, and Heilongjang, in China) for 2 years (2014 and 2015) with an average rate of more than 80% weed suppression. In addition, the BIO treatments significantly increased rice yield (16.3%-29.8% relative to the control) and yield components (e.g., number of spikes per square meter, plant height, and number of kernels per spike) (Li et al. 2018). However, the BIO effects on soil bacterial community, functional capability and soil chemical properties in multisite soil rice paddy are not well known. In this study, we evaluated: (1) the influence of BIO on soil bacterial community and soil enzyme and (2) the relationships among BIO, soil bacterial community and location (longitude and latitude). Results from this study lay a theoretical foundation for BIO applications.

## Materials and methods

### Bio-organic fertilizer (BIO) manufacturing

The organic substrates in the BIO consisted of kitchen garbage, maize straw, wood-destroying fungal dregs, rice straw, tobacco straw, plant ash, and chicken and sheep manure. The physical and chemical properties of the compost material are provided in our previous study (Li et al. 2018). The combined process of ZF-5.5 fertilizer mechine (Changsha Beye Agricultural Ltd, China) preparation and pile fermentation was used to produce composting manure under a temperature range of 40–80°C for 15 days. Man-made heating and cooling was used to control temperature on the first day. The compost was moved out form the ZF-5.5 fertilizer mechine and piled fermentation began one day later. After 15 days, the compost turned taupe gray, exhibited threadiness and had a slightly sour fragrance. This compost contained 53.4% organic matter, 2.0% N, 3.7% P_2_O_5_, and 1.1% K_2_O.

### Study site description

Our multi-site study was conducted in five rice paddies in China, which had already been in use for 30 years. The location, altitude, temperate climate, and growing season of these study sites have been given in Table 1.

**Table 1:** Description of the study sites

Site	Soil type	Latitude longitude	Altitude	Temperate climate	Growing season
Hainan (SY)	Latosol	N18°23′30″ E109°11′33″	9 m	22–28	April–November
Henan (HN)	Yellow Brown Soil	N 31°49′52″ E114°04′ 79″	87 m	25–34	April–October
Heilongjiang (HLJ)	Black Soil	N 45°97′82″ E128°75′40″	196 m	24–30	May–November
Jiangsu (JS)	Yellow Brown Soil	N 31°35′49″ E 119°10′57″	8 m	26–37	April–October
Guizhou (GZ)	Yellow Cinnamon Soil	N27°54′34″ E106°74′78″	1271 m	20–34	May–November

Soil types were classified according to China soil classification system (GB/T 17296-2009)

### Field experiment and soil sample collection

For each site, field trials were conducted on six 30 m × 20 m plots from April 25–27 to Oct. 18–23, 2018. Two or three 20-days old rice seedlings were transplanted to each hill per plot with 15 cm × 15 cm inter spacing seedlings in plot. The rice varieties selected were Chuanyou 6203 at Guizhou (GZ), Xiangeng 2369 at Henan (HN), Yuzhenxiang at Hainan sanya (SY), Longgeng 29 at Heilongjiang (HLJ) and Nangeng 9108 at Jianshu (JS). Three days after transplanting, BIO (3000 kg/ha) were spread over three plots as the treatment. Our choice of BIO dosage was based on results from our previous study which showed this dosage to be the most effective and economical for weed control (Li et al. 2018). The other plots were not supplied with BIO fertilizer. The base fertilizer was applied uniformly in six BIO-treatments and untreated plots. All field management practices followed local and traditional practices, except for the irrigation during BIO application, as a 3–5 cm water layer had to be maintained for 7 days. Rice plants were maintained as per site local agronomic practices. No top dressing or other weed control strategies were carried out in the experimental plots. Soil samples were collected from all plots on May 25–27, 2018, i.e. 1 month after BIO application. One hundred grams of surface soil (0–15 cm) was collected from 45 points and 15 samples were mixed together in 15 plastic bags from each plot. Soil samples were divided into two parts, one part was frozen and stored at − 80 °C, and the other part was air dried for 1 week and stored at 25 °C.

### Soil chemical properties measurements

Soil pH was measured in soil–water solution (W/V 1:5). Soil total N and K content were measured using an elemental analyzer (Carlo Erba, Milan, Italy) and total P content was assayed calorimetrically by the molybdate method (Willy et al. 2019). Other chemical properties which included hydrolytic N, extractable P, exchangeable K, and organic matter content were analyzed as described previously (Ballabio et al. 2019).

### DNA extraction and library construction

Total genomic DNA was extracted using DNA extraction kit following the manufacturer’s instructions. The quality and quantity of DNA was verified with NanoDrop and agarose gel. Extracted DNA was diluted to a concentration of 1 ng/μl and stored at − 20 °C until further processing. The diluted DNA was used as template for PCR amplification of bacterial 16S rRNA genes with the barcoded primers and Takara Ex Taq (Takara Bio, JP). For bacterial diversity analysis, V3V4 variable regions of 16S rRNA genes were amplified with the following primer pair: 343F-(5′-TACGGRAGGCAGCAG-3′) and 798R-(5′-AGGGTATCTAATCCT-3′).

Amplicon quality was visualized using gel electrophoresis, purified with AMPure XP beads (Bechman Couter, USA), and amplified for another round of PCR. After a second round of purification with the AMPure XP beads, the final amplicon was quantified using Qubit dsDNA assay kit (Takara Bio, JP). Equal amounts of purified amplicon were pooled for subsequent sequencing.

### Bioinformatics and statistical analysis

Raw sequencing data were in FASTQ format. Paired-end reads were then preprocessed using Trimmomatic software to detect and cut off ambiguous bases (N) (Tao et al. 2018). It also cut off low quality sequences with average quality score below 20 using the sliding window trimming approach. After trimming, the paired-end reads were assembled using the FLASH software (http://ccb.jhu.edu/software/FLSH/). Parameters of assembly were: 10 bp of minimal overlapping, 200 bp of maximum overlapping and 20% of maximum mismatch rate. Sequences were further performed with denoising as follows: reads with ambiguous, homologous sequences or below 200 bp were abandoned. Reads with 75% of bases above Q20 were retained. Then, reads with chimera were detected and removed. These two steps were achieved using QIIME software (version 1.8.0, http://qiime.org/).

Clean reads were subjected to primer sequences removal and clustering to generate operational taxonomic units (OTUs) using the V search software with 97% similarity cutoff. The representative read of each OTU was selected using QIIME package. All representative reads were annotated and blasted against the Silva database Version 123 (or Greengens) (16 s/18 s rDNA) using RDP classifier (confidence threshold was 70%). The alpha diversity indices (Chao1 and Shannon index) were calculated using QIIME in R software (Gomez-Sagasti et al. 2019). Principal component analysis (PCA) was performed using CANOCO 5.0. Analysis of variance (ANOVA) test was conducted with Genstat 13 (VSN International, Hemel Hemspstead, UK) to evaluate the effect of BIO treatment and control on the five sites. The relative abundance of OTU was inferred with FUNGuild (Wang et al. 2020). Significant differences in bacterial species were determined using the linear discriminant analysis (LDA) effect size (LEfSe) method with Kruskal–Wallis sumrank test. Differences were considered statistically significant at a level of *p* < 0.05. Phylogenetic analysis of communities by reconstruction of unobserved states (PICRUSt) analysis with the KEEG orthology database was used to predict and visualize bacterial function on the significant difference between bacteria. Redundancy analysis (RDA) was carried out in R, to determine the correlation among relative abundance of bacterial community, soil chemical properties, and site location.

### Soil enzymatic activity determination

Five soil samples from treated and untreated sites were analyzed for three representative enzymes activities (soil urease (S-UE), soil acid phosphatase (S-ACP) and soil β-glucosidase (S-β-GC). Soil enzyme activities were analysed with the S-UE, S-ACP, and S-β-GC assay kit (Solarbio life Sciences, Beijing, China). S-UE was defined as 1 g of soil which produced 1 μg NH_3_-N (U/g) daily. S-ACP was considered to be 1 g of soil which liberated 1 nmol phenol at 37℃ (U/g) daily. S-β-GC was considered to be 1 g of soil which produced 1 μmol p-nitroohenol (U/g) daily (Dick et al. 2013). All enzyme assays were conducted in duplicate.

## Results

### Effect of BIO on soil chemical properties

The effects of BIO on soil chemical properties in the five sites are presented in Table 2. No significant differences were observed between BIO treatment and untreated plots for soil pH, the total N, total K and total P at all five rice paddy sites. The hydrolytic N and organic matter of the soil with BIO treatment were respectively lower than for untreated soil at JS and HN sites. The other sites were not affected. The exchangeable K was higher in the BIO treatment (193 mg/kg and 243 mg/kg) than untreated plot (152 mg/kg and 142 mg/kg) at JS and GZ site rice paddy. The exchangeable K was not significantly different between treatments and control in the HLJ and SY sites. The exchangeable K of BIO soil was 23.66% lower than untreated soil. The exchangeable K was irregularly affected by BIO at the five sites. The extractable P also showed irregularity in its influence by BIO in the five rice paddy sites.

**Table 2:** Chemical properties of the surface soil layer (0–15 cm) with the BIO-treatment and control in five sites

Variable source	pH	Total K mg/kg	Total N mg/kg	Total P mg/kg	Exchangeable K mg/kg	Extractable P mg/kg	Hydrolytic N mg/kg	Organic matter mg/kg
HN-CK	5.42a	17.9a	2.33a	0.42a	*131a*	0.26a	*233a*	*34.1a*
HN-TRE	5.33a	17.8a	1.55b	0.36a	*100b*	0.26a	*161b*	*26.1b*
JS-CK	6.13a	13.4a	1.37a	0.46a	*152b*	*2.26a*	*174a*	*18.8a*
JS-TRE	6.60a	14.7a	1.15a	0.50a	*193a*	*1.87b*	*120b*	*14.8b*
HLJ-CK	5.54a	21.8a	1.70a	0.82a	131a	*1.40b*	179a	34.0a
HLJ-TRE	5.73a	21.7a	1.73a	1.00a	128a	*8.91a*	150a	32.1a
GZ-CK	5.94a	15.3a	2.16a	0.91a	*142b*	4.25a	180a	34.3a
GZ-TRE	6.47a	16.7a	2.23a	1.06a	*243a*	5.30a	182a	36.6a
SY-CK	6.30a	*10.0b*	1.03a	0.41a	121a	2.17a	155a	15.5a
SY-TRE	6.35a	*13.1a*	1.12a	0.34a	128a	2.01a	117a	16.1a

Italic values represent *p* < 0.05

Values show mean (n = 3). Means with different letters represent significant differences at *p* < 0.05

### Composition of soil bacterial community at the study sites

**Fig. 1:**
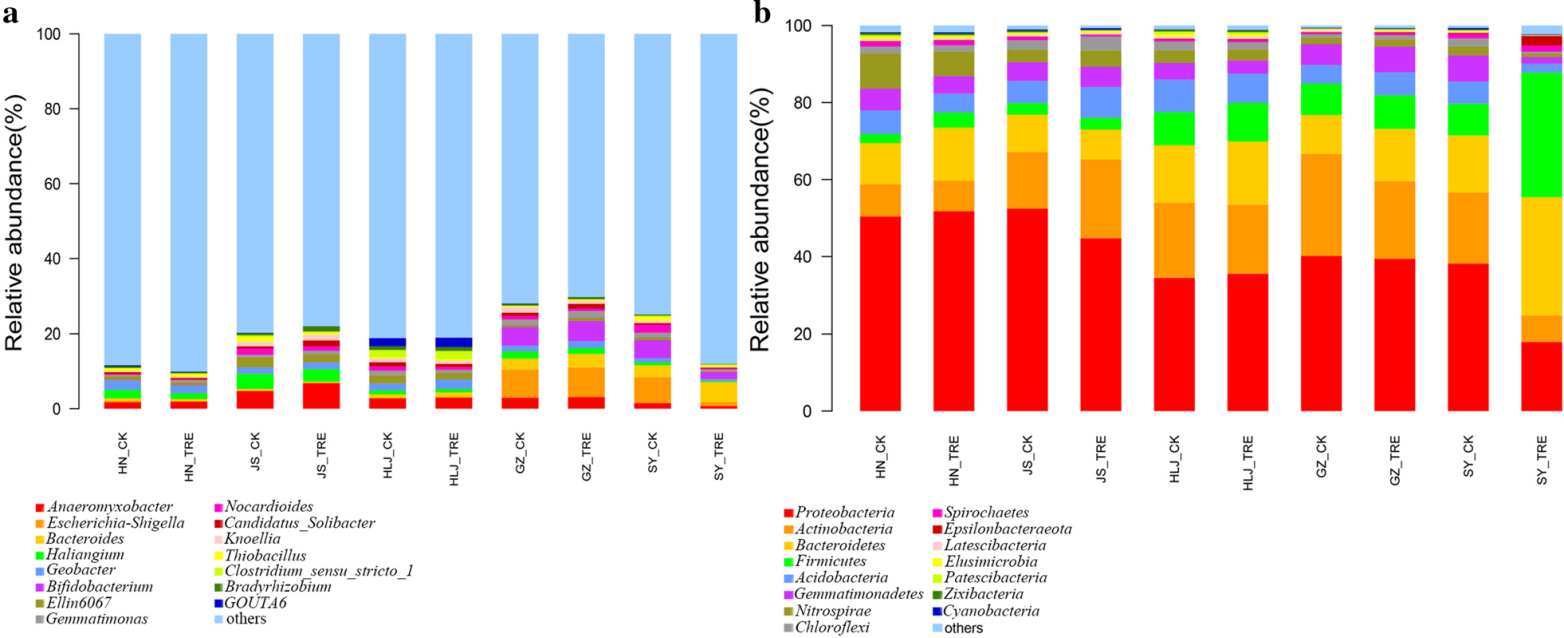
Relative abundances (%) of bacterial composition (phyla and genera level) in BIO-treated and untreated soil samples at five rice paddy sites. **a** Relative abundance of the top 15 phyla among the BIO-treated soil samples. **b** Relative abundance of the top 15 genera among the BIO-treated soil samples. The HN_CK, HN_TRE, JS_CK, JS_TRE, HLJ_CK, HLJ_TRE, GZ_CK, GZ_TRE, SY_CK and SY_TRE represent BIO treated and untreated control at the five rice paddy sites in China

Sequencing of the 16S rRNA genes revealed the bacterial diversity and community composition in the five sites of the rice paddies. The number of OTUs in all samples was 1660-4621 and the mean length of valid tags was 409.98-419.42 bp in all samples (Additional file 1: Fig. S1). Bacterial community structure at the phylum level in the BIO-treated soil and untreated soil samples is shown in Fig. 1a. The five most dominant phyla in HN soil samples were *Proteobacteria*, *Bacteroidetes*, *Actinobacteria*, *Nitrospirae*, and *Acidobacteria*. The five most dominant phyla in JS soil samples were *Proteobacteria*, *Actinobacteria*, *Bacteroidetes*, *Acidobacteria*, and *Gemmatimonadetes*. The five most dominant phyla were *Proteobacteria*, *Bacteroidetes*, *Actinobacteria*, *Firmicutes*, and *Acidobacteria* in HLJ, GZ, and SY soil samples. Bacterial community structure (at the genus level) in the BIO-treated and untreated soil samples is shown in Fig. 1b. The five most dominant genuses in HN soil samples were *Anaeromyxobacter*, *Haliangium*, *Geobacter*, *Ellin6067*, and *Thiobacillus*. The five most dominant genus in JS soil samples were *Anaeromyxobacter*, *Haliangium*, *Ellin6067*, *Geobacter*, and *Nocardioides*. The five most dominant genus in HLJ soil samples were *Anaeromyxobacter*, *Gouta6*, *Geobacter*, *Clostridium_ sensu_stricto_1*, and *Ellin6067*. The five most dominant phyla in GZ soil samples were *Escherichia-Shigella*, *Bifidobacterium*, *Anaeromyxobacter*, *Bacteroides* and *Haliangium*. The five most dominant genus in SY soil samples were *Escherichia-Shigella*, *Bacteroides*, *Bifidobacterium*, *Anaeromyxobacter*, and *Nocardioides*. Hence, the main phyla were *Proteobacteria*, *Actinobacteria*, *Bacteroidetes*, *Firmicutes* and *Acidobacteria*, and the main genus were *Anaeromyxobacter*, *Escherichia-Shigella*, *Bacteroides*, *Haliangium*, and *Geobacter* in the five rice paddies.

### Soil bacterial community diversity of the study sites

**Fig. 2:**
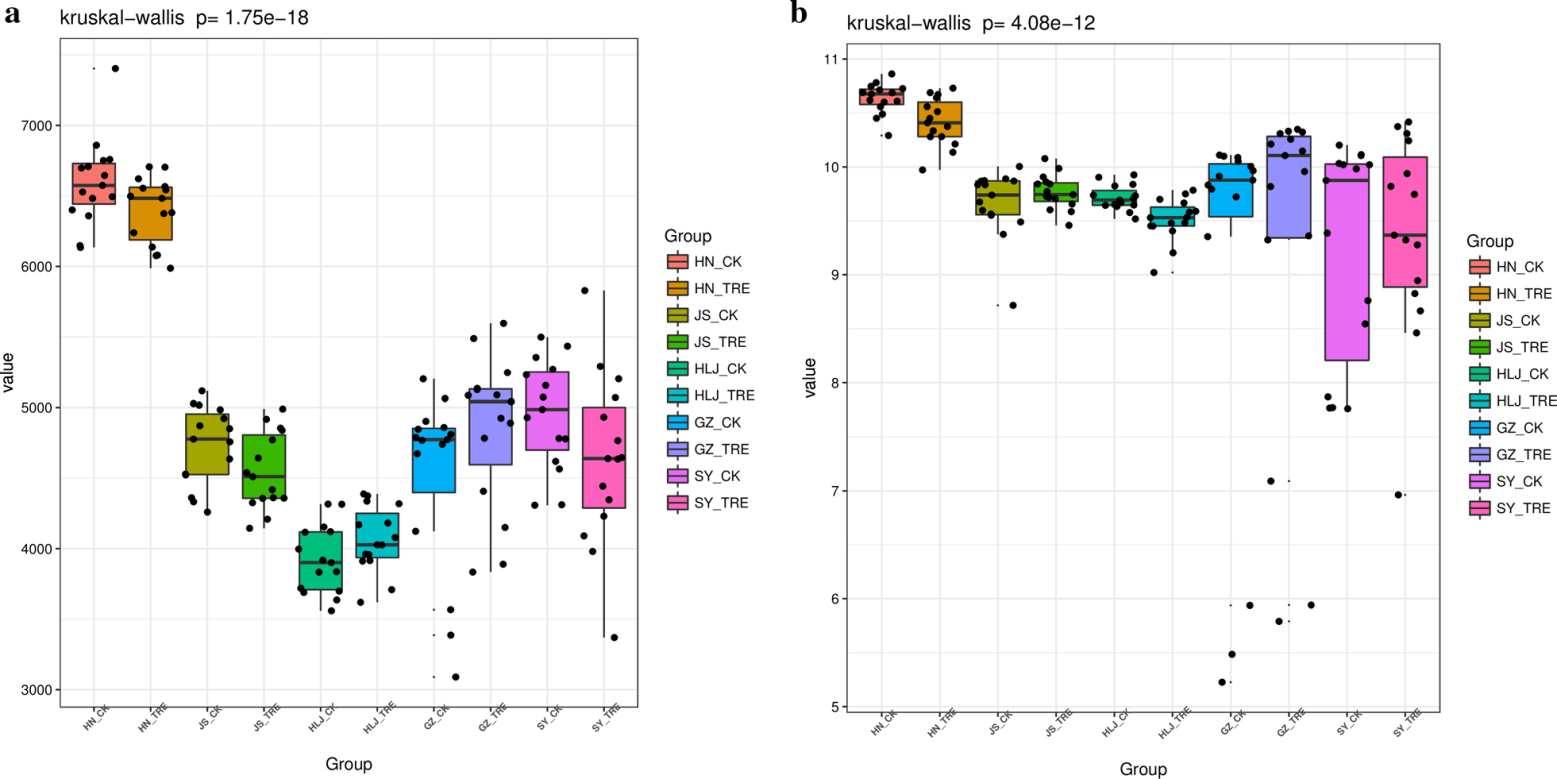
Alpha-diversity indices, **a** Chao1 and **b** Shannon indices, of the bacterial community structure in BIO-treated and untreated soil samples at five rice paddy sites. The HN_CK, HN_TRE, JS_CK, JS_TRE, HLJ_CK, HLJ_TRE, GZ_CK, GZ_TRE, SY_CK and SY_TRE represent BIO treatment and untreated control at the five rice paddy sites in China

**Fig. 3:**
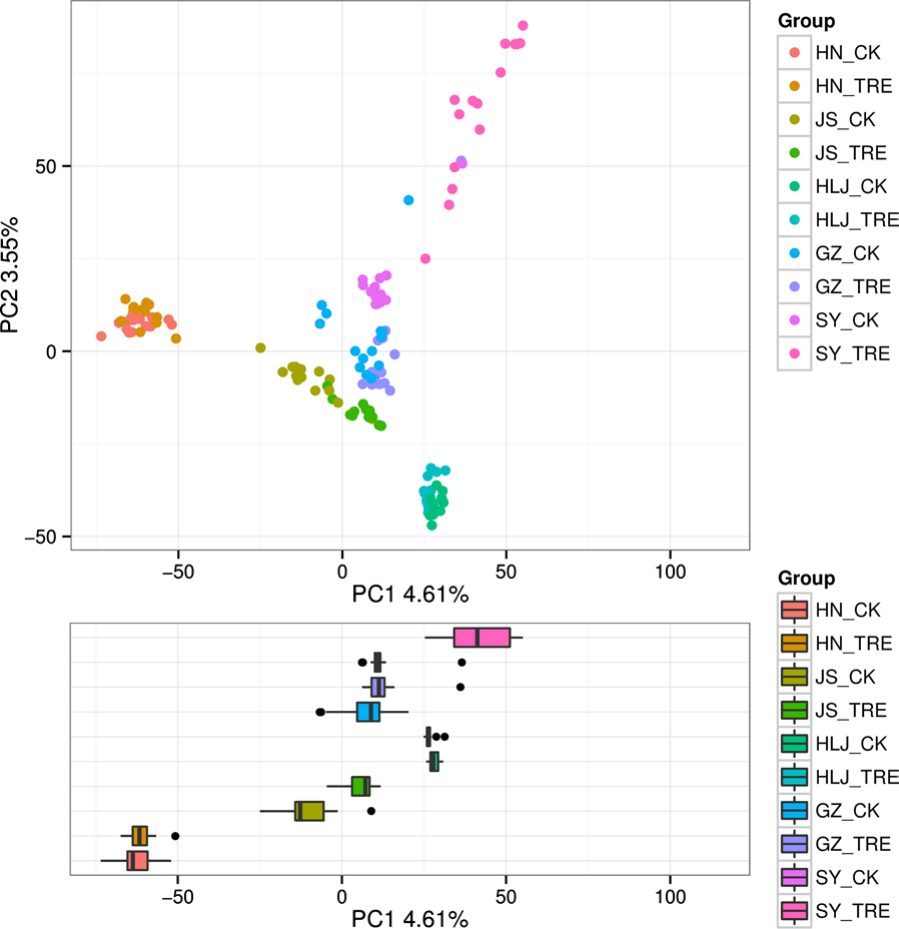
Beta-diversity indices (PCA plot) of bacterial community structure in BIO-treated and untreated soil samples at five rice paddy sites. The HN_CK, HN_TRE, JS_CK, JS_TRE, HLJ_CK, HLJ_TRE, GZ_CK, GZ_TRE, SY_CK and SY_TRE represent BIO treatment and untreated control at the five rice paddy sites in China

The alpha diversity indices (Chao1and Shannon) of the different soil treatments (BIO-treatment and untreated control) are shown in Fig. 2. The Chao1 indices indicated that any soil treatments of one site was not significantly separated (*p* < 0.05, Fig. 2a) and the Shannon indices also indicated d that the overall bacterial species were not significantly separated (*p* < 0.05, Fig. 2b) among the BIO-treated and untreated soil samples at any one site. The indices were not significantly different among the BIO-treated and untreated soil samples. The PCA of beta diversity indicated that all replicates of treated soils (BIO samples and untreated soil samples) clustered together (Fig. 3). The soil treatments of HN, HLJ, and JS site was not significantly separated (*p* < 0.05) and the GZ and SY also verified that the overall bacterial species were significantly separated (*p* < 0.05) among the BIO-treated and untreated soil samples at any one site. The PCA of beta diversity showed that the BIO treatment had minor influence on the beta diversity.

### Changes in the soil bacterial community

**Fig. 4:**
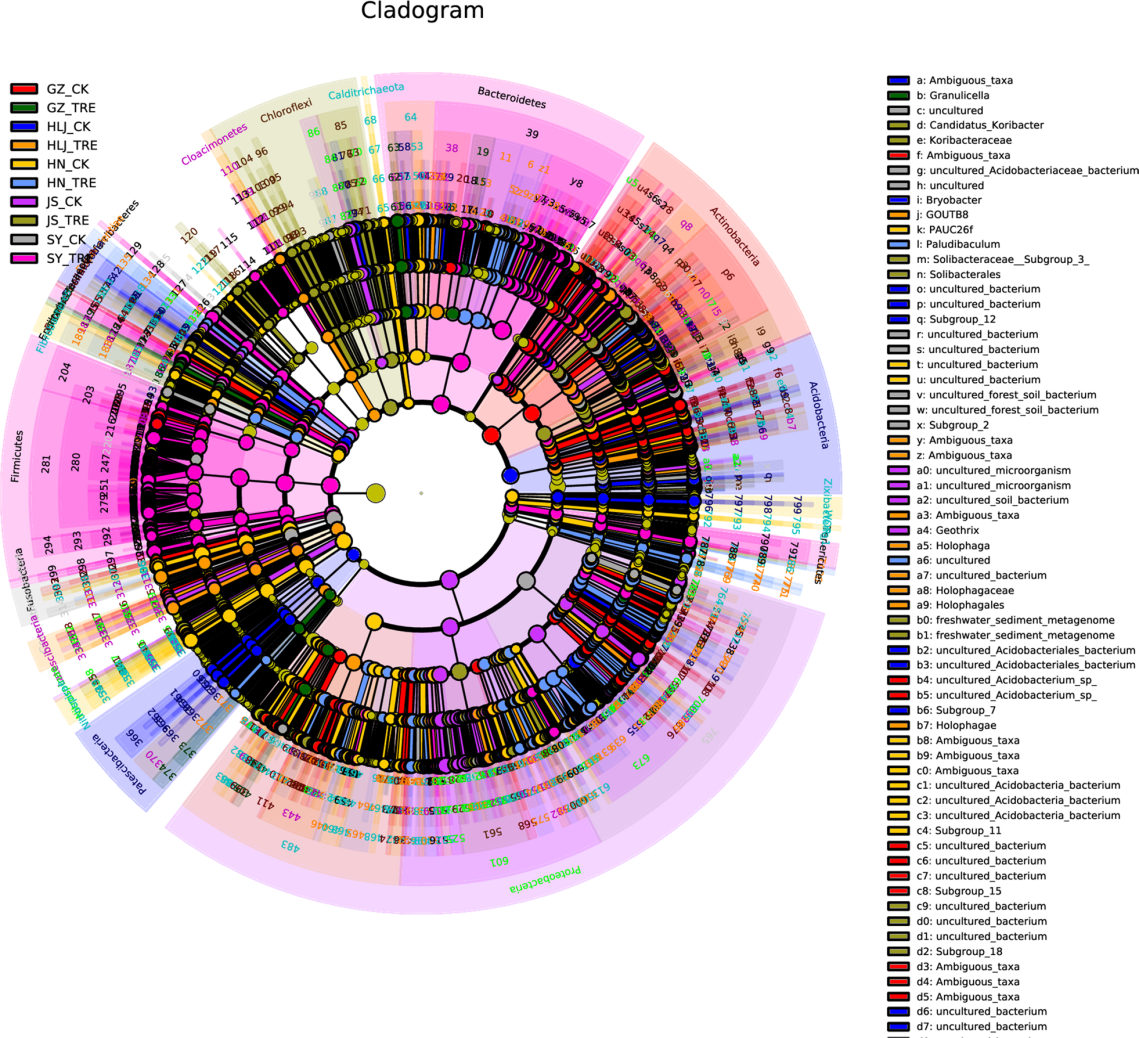
Linear discriminant analysis (LDA) effect size (LEfSe) analysis on the different biomarkers between BIO-treated and untreated soils at five rice paddy sites. Biomarkers that are significantly associated with each treatment, with LDA scores larger than 2 are shown. Significantly discriminant taxon nodes are colored. Each circle’s diameter is proportional to the taxon’s abundance. Labels are shown of the phylum, class and order levels. The LDA scores of each identified biomarker from the phylum to genus levels are shown in Additional file 1: Fig. S2. The HN_CK, HN_TRE, JS_CK, JS_TRE, HLJ_CK, HLJ_TRE, GZ_CK, GZ_TRE, SY_CK and SY_TRE represent BIO treated and untreated soil samples at the five rice paddy sites in China

To investigate the effects of BIO on the changes in soil bacterial community, ANOVA test was used to identify differential abundance between BIO-treatment and untreated control in five site soils (Additional file 1: Fig. S1). At the phylum level, the top 10 significantly differences abundant bacteria were *Acidobacteria*, *Actinobacteria*, *Bacteroidetes*, *Chloroflexi*, *Epsilonbacteraeota*, *Firmicutes*, *Gemmatimonadetes*, *Nitrospirae*, *Proteobacteria* and *Spirochaetes*. At the genus level, the significantly difference bacteria were *Anaeromyxobacter*, *Bacteroides*, *Bifidobacterium*, *Escgerichia-Shigella*, *Geobacter*, and *Haliangium*. LEfSe analysis identified 47 differentially abundant phyla and 963 differentially abundant genus with LDA scores > 2 (Fig. 4). There were 12 phyla in SY_CK and SY_TR with LDA scores > 5. The most significant contribution of BIO on the soil community in the SY site was the enhancement of the abundance of *Gammaproteobacteria* (at phylum level). In addition, enhancement of the abundance of *Proteobacteria* and *Deltaproteobacteria* were the major effects BIO had on the soil community in JS_CK and JS_TR. Of the 15 phyla in HN_CK and HN_TR with LDA scores > 4, the key contributor of BIO effect on soil community were *Alphaproteobacteria* and *Nitrospirae*. Nine phyla in HLJ and 7 phylum in GZ with LDA scores > 4, the effect BIO had on the soil community, was the enhancement of the abundance of *Acidobacteria* and *Actinobacteria*, respectively.

**Fig. 5:**
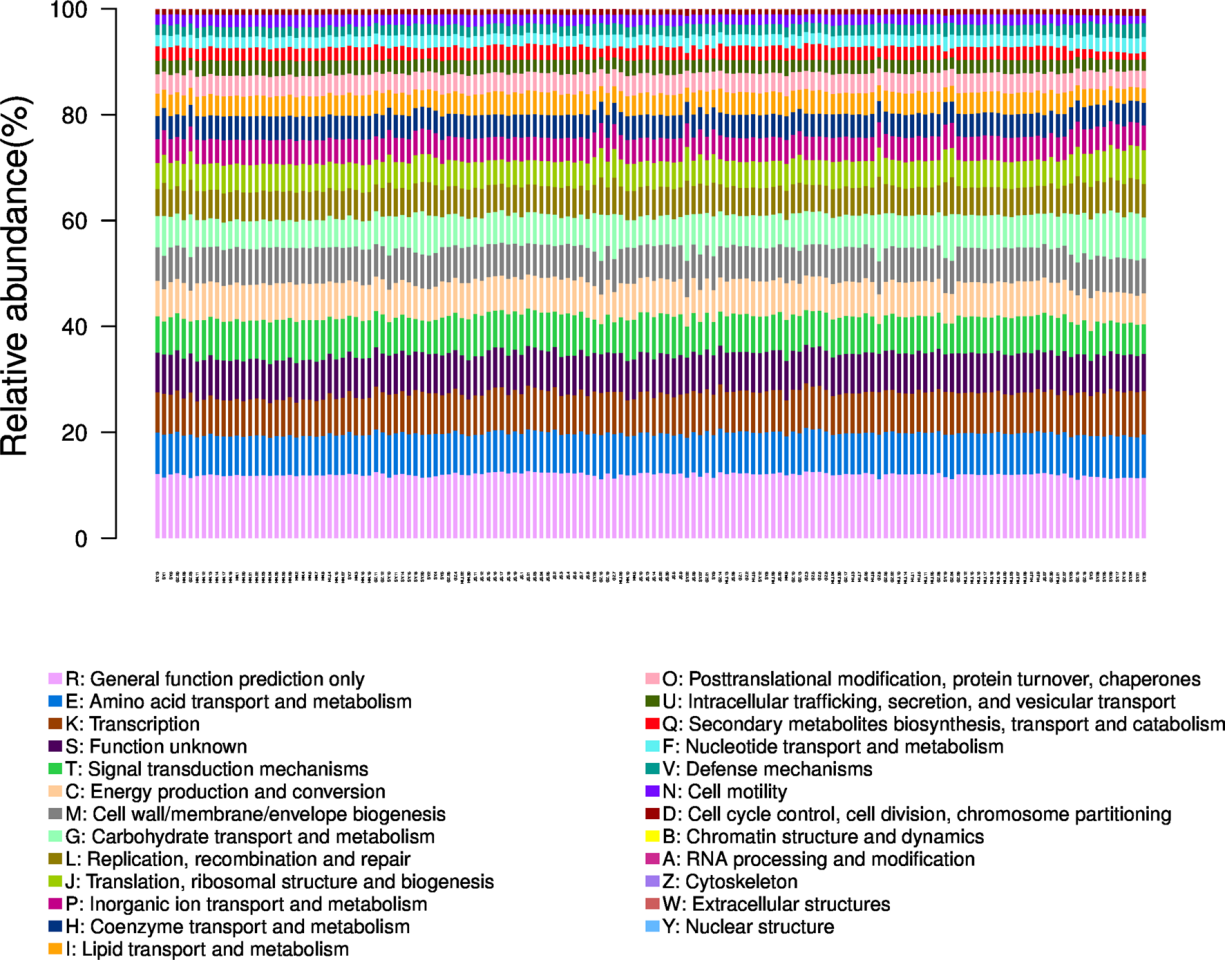
Clusters of orthologous groups of proteins (COG) functional prediction of the significantly different abundant bacteria between BIO-treated and untreated soil samples at five rice paddy sites. The HN_CK, HN_TRE, JS_CK, JS_TRE, HLJ_CK, HLJ_TRE, GZ_CK, GZ_TRE, SY_CK and SY_TRE represent BIO treatedt and untreated soil samples at the five rice paddy sites in China

Based on the KEEG data, 6 functional gene families were predominant and accounted for metabolism (50.69%), genetic information processing (16.29%) and environmental information processing (13.25%), at KEGG level 1 (Additional file 1: Fig. S2). At KEGG level 2, a total of 41 sub-functional gene families were identified to be involved in cell communication, sensory system and amino acid metabolism (Additional file 1: Fig. S3). In brief, only cell communication (*p* > 0.01) and sensory system (*p* > 0.01) were significantly reduced in all study sites. The other 39 sub-functional gene families were not concurrently affected at all the study sites. From baslt EggNOG data, a total of 25 functional clusters of COG (Clusters of Orthologous Groups, https://www.ncbi.nlm.nih.gov/COG/) were predicted in all sites (Fig. 5). In brief, the relative abundance of COG gene was enhanced by the BIO treatment in all sites. The top 3 functional clusters were general function prediction only (R), amino acid transport and metabolism (E) and transcription (K) in all study sites. The abundance of genes related to COG was similar between BIO treatments and controls at the multisites in China rice paddies.

### Relationships of the bacterial community with soil chemical properties and site locations

**Fig. 6:**
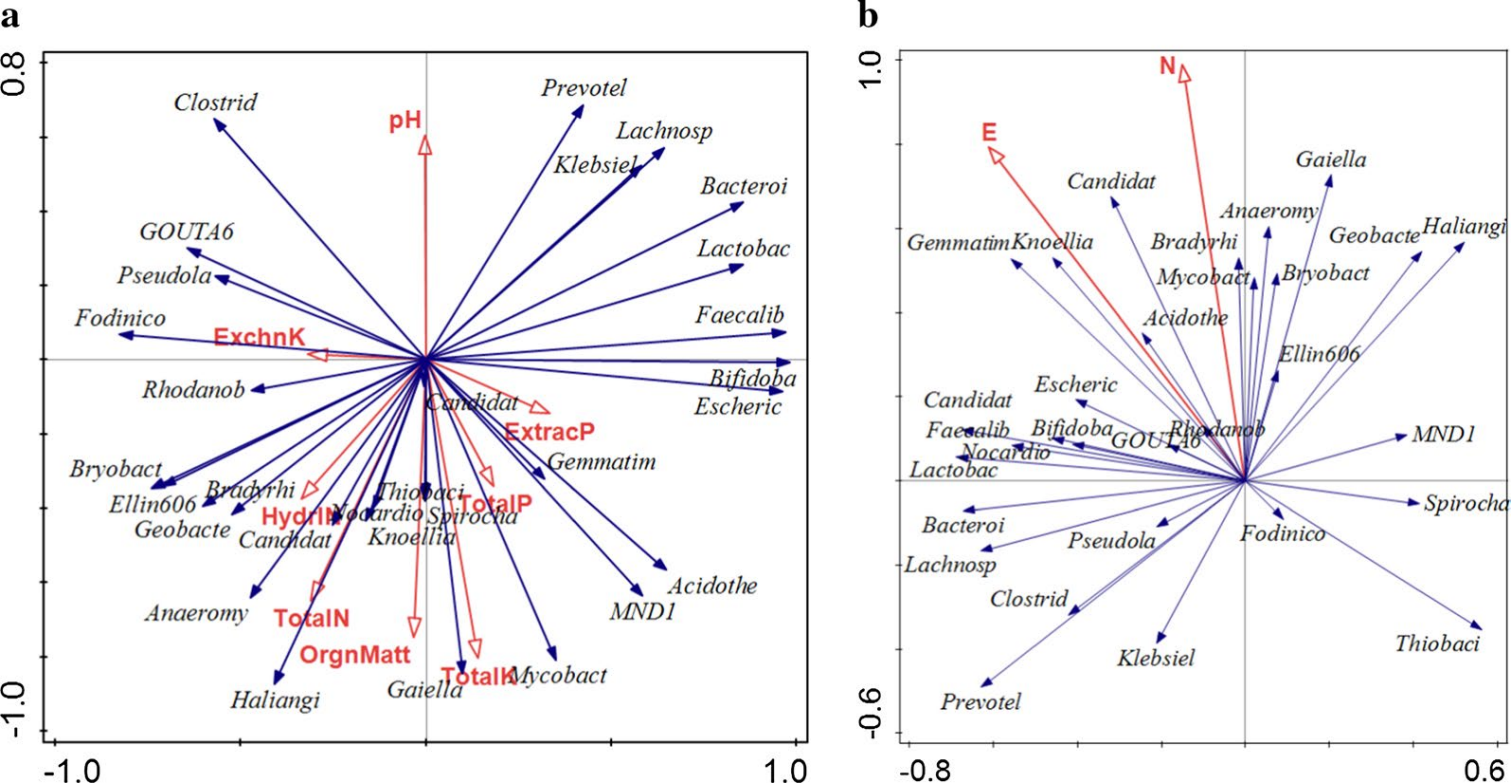
Redundancy analysis (RDA) of microbial community and soil chemical properties (*p* value = 0.02) between BIO-treated and untreated soil samples at five rice paddy sites. E: East longitude, N: Northern latitude, ExchnK: Exchangeable K, HydrIN: Hydrolytic N, ExtracP: Extractable P

The correlation among bacterial community, soil chemical properties, and site locations is shown in Fig. 6 and Table 3. We observed significant positive correlations among northern latitude, eastern longitude, exchangeable K, total K, total P, soil pH, and total N in our study, except for organic matter, hydrolytic N and extractable P. The northern latitude and eastern longitude of rice paddy sites were positively related to the abundance of *Escherichia.Shigella* (*p* = 0.022 and 0.001), *Bifidobacterium* (*p* = 0.007 and 0.001), *Ellin6067* (*p* = 0.007 and 0.006), *GOUTA6* (*p* = 0.006 and 0.046), *Pesudolabrys (p* = 0.039 and 0.010), *Acidothermus* (*p* = 0.030 and 0.036), *Klebsiella* (*p* = 0.069 and 0.069) and *Fodinicola* (*p* = 0.002 and 0.001). In addition, exchangeable K significantly correlated with *Anaeromyxobacter* (*p* = 0.001) *Candidatus_Solibacter* (*p* = 0.004), Knoellia (*p* = 0.021), *Bradyrhizobium* (*p* = 0.028) and *Candidatus_ Koribacter* (*p* = 0.047).

**Table 3:** Canonical correlation analysis among soil bacterial, soil chemical properties and soil sites

Bacterial genus	Organic matter	Hydrolytic N	Exchangeable K	pH	Total N	Total P	Total K	N	E	Extractable P
*Anaeromyxobacter*	0.865	0.838	***0.001***	0.331	0.560	***0.044***	0.657	0.216	0.407	0.401
*Escherichia.Shigella*	0.527	0.418	0.116	***0.037***	0.243	0.360	0.200	***0.022***	***0.001***	0.231
*Geobacter*	0.178	0.204	0.828	0.096	***0.024***	0.367	***0.003***	***0.026***	0.260	0.602
*Bifidobacterium*	0.178	0.204	0.894	0.682	0.537	0.946	0.313	***0.007***	***0.001***	0.498
*Ellin6067*	0.892	0.707	0.206	0.811	0.682	0.279	0.279	***0.007***	***0.006***	0.881
*Gemmatimonas*	0.313	0.349	0.166	0.331	0.448	***0.035***	0.973	0.702	0.367	0.121
*Candidatus_Solibacter*	0.427	0.470	***0.004***	0.296	0.279	***0.014***	0.407	0.298	0.634	0.383
*Knoellia*	1	0.838	***0.021***	0.407	0.759	0.073	0.919	0.335	0.449	0.336
*Bradyrhizobium*	0.892	1	***0.028***	0.707	0.492	***0.039***	0.178	0.016	0.046	0.455
*GOUTA6*	0.178	0.263	0.947	***0.014***	0.066	0.232	***0.001***	***0.006***	***0.046***	0.841
*Bryobacter*	0.492	0.537	0.089	1	0.218	***0.049***	***0.049***	***0.009***	0.058	0.894
*Candidatus_Koribacter*	0.733	0.514	***0.047***	0.060	0.838	0.073	0.919	0.576	0.492	0.132
*Pesudolabrys*	0.583	0.865	0.828	0.514	0.973	0.584	0.232	***0.039***	***0.010***	0.777
*Acidothermus*	0.919	0.811	0.185	***0.010***	0.838	0.838	0.080	***0.030***	***0.036***	0.614
*Klebsiella*	0.891	0.759	0.424	0.448	0.707	0.367	0.232	***0.069***	***0.069***	0.700
*Fodinicola*	0.492	0.560	0.815	0.514	0.946	0.448	0.154	***0.002***	***0.001***	0.960

N: Northern latitude; E: East longitude; Boldface: significance; Italic boldface: top effect on soil chemical properties and soil sites

### Soil enzymatic activity

**Fig. 7:**
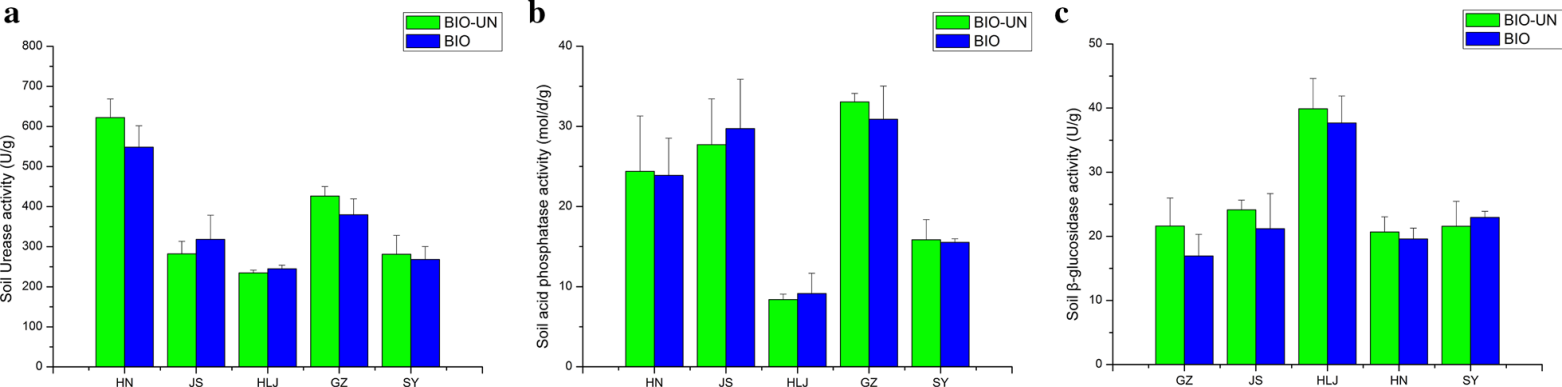
Soil enzyme activity in the BIO-treated and untreated soil at five site soils. **a** soil urease, **b** soil acid phosphatase, **c** soil β-glucosidase. Vertical bars indicate standard deviation of the mean (n = 3). For each parameter, different letters indicate significant differences between means at *p* < 0.05. The HN, JS, HLJ, GZ and SY represent BIO treated and untreated soil samples at the five rice paddy sites in China

The activities of three soil enzymes (S-UE, S-ACP and S-β-GC) in the BIO-treated and untreated soil at five sites were not significantly different (Fig. 7). S-UE and S-ACP activity in BIO treated soil decreased when compared to its activities in untreated soil at the GZ, SY, HN sites of rice paddies. On the contrary, S-UE and S-ACP exhibited opposite activity in the other sites (JS and HLJ). However, the BIO treated soil experienced decreased S-β-GC activity when compared to untreated soil at GZ, JS, HLJ, and HN sites except for the SY sites. In majority of the sites, the three enzyme activities under BIO-treatment were equivalent to that under untreated.

## Discussion

In this study, we identified the main bacterial phyla (*Proteobacteria*, *Actinobacteria*, *Bacteroidetes*, *Firmicutes* and *Acidobacteria*) and genus (*Anaeromyxobacter*, *Escherichia-Shigella*, *Bacteroides*, *Haliangium*, and *Geobacter*) in five rice paddies in China. *Proteobacteria* was relatively the most abundant phylum in the soils of the five rice paddies located in Guizhou province (Southwestern China), and the other four phyla (*Chloroflexi*, *Acidobacteria*, *Nitrospirae*, and *Bacteroidetes*) were the dominant species in all samples. This result is consistent with that of a previous study (Sun et al. 2015). Results from the taxonomic analysis indicated that the *Cyanobacteria* and *Proteobacteria* were dominant phyla in four soils, collected from Changchun, Jiangdu, Yingtan, and Yanting rice paddies (Wang et al. 2019a, b). At Xiantang (Hunan Province, China), the dominant phyla were *Proteobacteria* (39.98%), *Chloroflexi* (17.10%) and *Actinobacteria* (12.70%) out of the total 41 phyla of bacterial community structure in the rice paddy (Guo et al. 2019). At Hengyang (Hunan Province, China), the *Proteobacteria*, *Acidobacteria*, *Nitrospirae*, *Gemmatimonadetes*, and *Verruco- microbia* were the dominant phyla of bacterial community structure in three rice-based cropping paddies (Huang et al. 2020). From the above reports, we deduce that the dominant soil bacterial species were similar in all the major rice-growing areas in China. Hence, application of BIO did not change the soil bacterial structure within these rice paddies.

It is generally accepted that farming practices cause changes in the soil microbial community (Zwetsloot et al. 2020). Likewise, BIO application caused changes in the soil microbial communities in all the five rice paddies and the dominant and most abundant bacteria present included *Gammaproteo-bacteria*, *Proteobacteria*, *Deltaproteobacteria*, *Alphaproteobacteria, Nitrospirae*, *Acidobacteria*, and *Actinobacteria*. Meanwhile, the top three functions of the bacteria in all study sites were only of general function prediction, amino acid transport and metabolism and transcription. Land-use changes of desert soils resulted into a significant decrease in *Alphaproteobacteria*, *Actinodbacteria*, *Bacteroidetes* and *Firmicutes* and sharply increased *Acidobacteria*, *Chlorflexi*, *Nitrospira* and *Gammaproteobacteria* (Wang et al. 2012). The phylum *Bacteroidetes* and *Acidobacteria* were increased, while *Actinobacteria* and *Firmicutes* decreased under combined antibiotics (sulfadiazine, sulfamethoxazole, trimethoprim, florfenicol, and clarithromycin) treatment in rice system (Uddin et al. 2019). Amino acid transport and metabolism was significantly different among the soils in the paddies under four common fertilizer treatment and control without fertilizer treatment (Wang et al. 2019a, b). Similar to these studies, soil bacterial community and function were slightly different between application of BIO and untreated plots in the five rice paddies.

Farming practices commonly cause changes in soil chemical properties and enzyme activity (Kumar et al. 2020). However, there were minor modifications after application of BIO in the five rice paddies (Table 2, Fig. 7). This result is in agreement with other studies. For instance, soil chemical properties and enzyme activity differed significantly between the organic site and conventional site, while no changes were stimulated in response to lupin (*Lupinus angustifolius*) amendment at 4 and 8 t level during short-term incubation (Stark et al. 2008). Also, the soil properties at a rice-rice rotation producing area in china did not show differences between optimum reduced fertilization (OPT) treatments and unfertilized control (CK) and the soil pH, total phosphorus, and total potassium showed no significant differences among all treatments under a long-term fertilization field experiment at rice-rice rotation producing area in China (Zhu et al. 2019). The activities of urease and acid phosphatase in the mesotrione-treated and control soil were not different from 2nd to 20th day after application (Du et al. 2018). The silver nanoparticles (AgNPs) had minor influence on the soil physico-chemical properties and enzyme activities (Oca-Vasquez et al. 2020). Similarly, the application of BIO in our study did not change the main soil chemical properties and enzyme activity in China rice paddies.

The present study demonstrated how BIO influenced bacterial community, soil enzyme and soil chemical properties. Application of BIO did not change the main soil bacterial phyla and genus, but resulted in a slightly different bacterial community and minor modification of soil enzyme and chemical properties in the rice paddies. These finding suggests that BIO application may be a sustainable weed management strategy in rice system. Future research under long-term field studies at multiple sites in rice paddies will shed more light on our findings.

## Supplementary Information

**Additional file 1: Figure S1.** The number of OTUs and mean length of valid tags between BIO treated and untreated in five sites. The HN_CK, HN_TRE, JS_CK, JS_TRE, HLJ_CK, HLJ_TRE, GZ_CK, GZ_TRE, SY_CK and SY_TRE represent BIO treated and untreated control at the five rice paddy sites in China. **Figure S2.** KEGG level 1 analysis sub-functional gene families between BIO treated and untreated in five sites. The HN_CK, HN_TRE, JS_CK, JS_TRE, HLJ_CK, HLJ_TRE, GZ_CK, GZ_TRE, SY_CK and SY_TRE represent BIO treated and untreated control at the five rice paddy sites in China. **Figure S3.** KEGG level 2 analysis sub-functional gene families between BIO treated and untreated in five sites. The HN_CK, HN_TRE, JS_CK, JS_TRE, HLJ_CK, HLJ_TRE, GZ_CK, GZ_TRE, SY_CK and SY_TRE represent BIO treated and untreated control at the five rice paddy sites in China.

## Data Availability

The datasets generated during and/or analysed during the current study are available in the NCBI, https://submit.ncbi.nlm.nih.gov/subs/sra/SUB8184095/overview. The associated BioProject number is PRJNA691100. The associated SRA numbers are SRR13413673-SR R13413682, respectively.

## Abbreviations

BIO: Novel bioorganic fertilizer
CBF: Common fertilizer
RDA: Redundancy analysis
PCA: Principal coordinates analysis
EF: Control effect
