# Supplementary material for: Effects of novel bioorganic fertilizer application on soil enzymes and bacterial community in multi-site rice paddies in China
Source: AMB Express. 2021 May 31;11:79. doi: 10.1186/s13568-021-01241-5 (PMC8167081; doi:10.1186/s13568-021-01241-5)
Supplement: Supplementary file 1 — Additional file 1: Figure S1. The number of OTUs and mean length of valid tags between BIO treated and untreated in five sites. The HN_CK, HN_TRE, JS_CK, JS_TRE, HLJ_CK, HLJ_TRE, GZ_CK, GZ_TRE, SY_CK and SY_TRE represent BIO treated and untreated control at the five rice paddy sites in China. Figure S2. KEGG level 1 analysis sub-functional gene families between BIO treated and untreated in five sites. The HN_CK, HN_TRE, JS_CK, JS_TRE, HLJ_CK, HLJ_TRE, GZ_CK, GZ_TRE, SY_CK and SY_TRE represent BIO treated and untreated control at the five rice paddy sites in China. Figure S3. KEGG level 2 analysis sub-functional gene families between BIO treated and untreated in five sites. The HN_CK, HN_TRE, JS_CK, JS_TRE, HLJ_CK, HLJ_TRE, GZ_CK, GZ_TRE, SY_CK and SY_TRE represent BIO treated and untreated control at the five rice paddy sites in China. [file 13568_2021_1241_MOESM1_ESM.docx]

**AMB Express**

Effects of novel bioorganic fertilizer application on soil enzymes and bacterial community in multi-site rice paddies in China

Zuren Li, Jingcai Han, Haodong Bai, Di Peng, Lifeng Wang, Lianyang Bai*

State key laboratory of hybrid rice, Hunan Academy of Agricultural Sciences, Changsha 410125, P.R. China

Corresponding authors ^*^E-mail: lybai196712@163.com. Phone: +86-0371-84696075. Fax number: +86-0371-84696025.

**Figure Legends**

Figure S1. The number of OTUs and mean length of valid tags between BIO treated and untreated in five sites.

The HN_CK, HN_TRE, JS_CK, JS_TRE, HLJ_CK, HLJ_TRE, GZ_CK, GZ_TRE, SY_CK and SY_TRE represent BIO treated and untreated control at the five rice paddy sites in China.

Figure S2. KEGG level 1 analysis sub-functional gene families between BIO treated and untreated in five sites.

The HN_CK, HN_TRE, JS_CK, JS_TRE, HLJ_CK, HLJ_TRE, GZ_CK, GZ_TRE, SY_CK and SY_TRE represent BIO treated and untreated control at the five rice paddy sites in China.

Figure S3. KEGG level 2 analysis sub-functional gene families between BIO treated and untreated in five sites.

The HN_CK, HN_TRE, JS_CK, JS_TRE, HLJ_CK, HLJ_TRE, GZ_CK, GZ_TRE, SY_CK and SY_TRE represent BIO treated and untreated control at the five rice paddy sites in China.


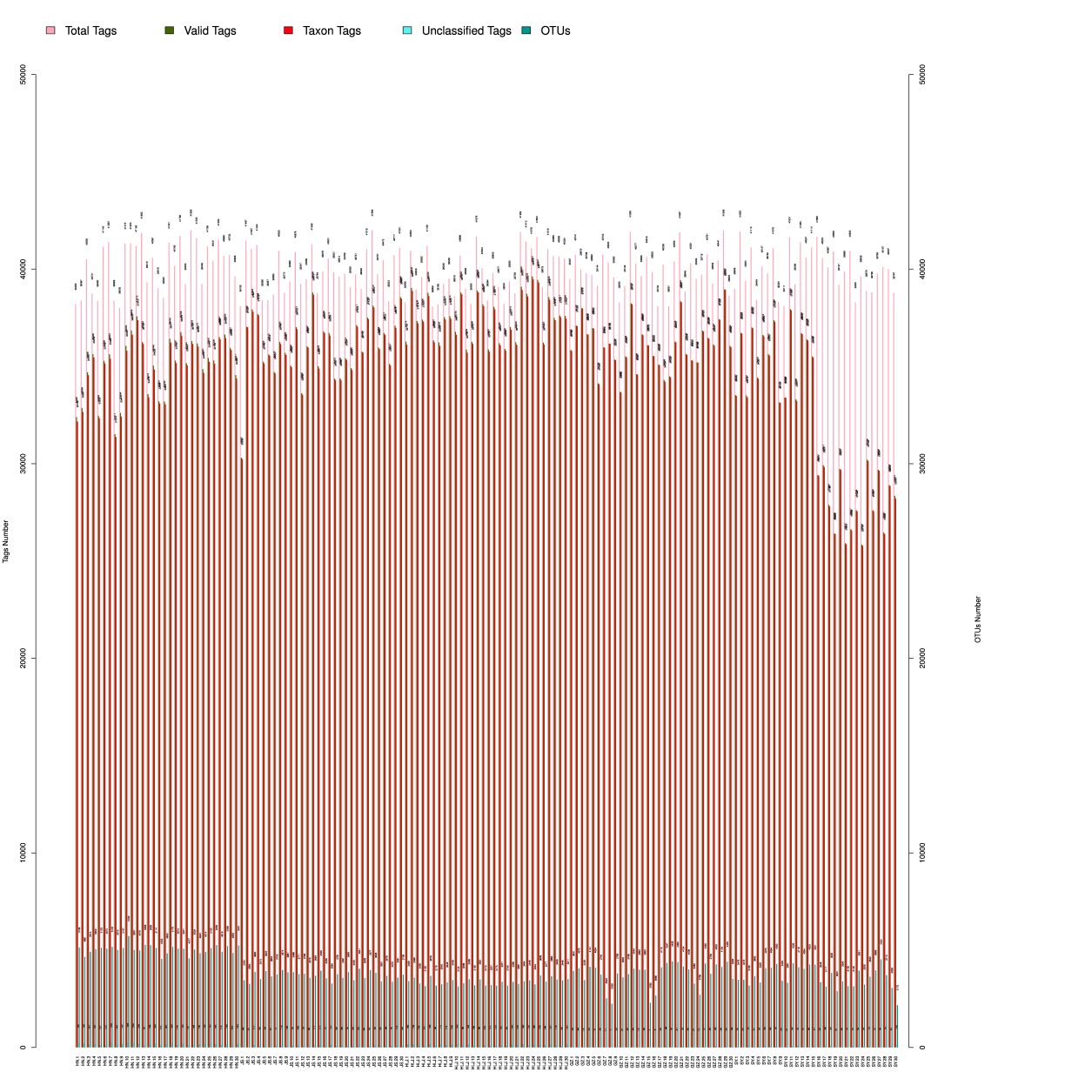
Figure S1.


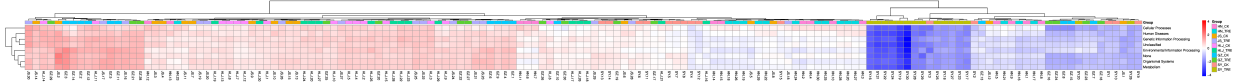


Figure S2


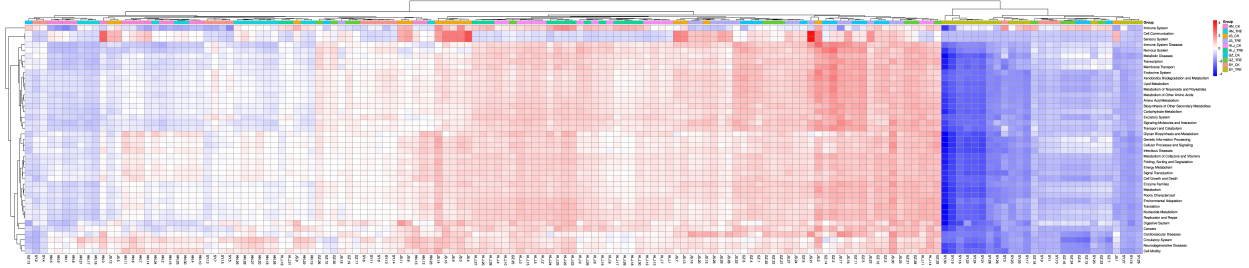


Figure S3.
